# Comparative Physiological and Molecular Analyses of Two Contrasting Flue-Cured Tobacco Genotypes under Progressive Drought Stress

**DOI:** 10.3389/fpls.2017.00827

**Published:** 2017-05-17

**Authors:** Xinhong Su, Fengjie Wei, Yongjin Huo, Zongliang Xia

**Affiliations:** ^1^College of Life Science, Henan Agricultural UniversityZhengzhou, China; ^2^Henan Institute of Tobacco ScienceZhengzhou, China

**Keywords:** tobacco, drought, antioxidant enzymes, gene expression, ROS

## Abstract

Drought is a major environmental factor that limits crop growth and productivity. Flue-cured tobacco (*Nicotiana tabacum*) is one of the most important commercial crops worldwide and its productivity is vulnerable to drought. However, comparative analyses of physiological, biochemical and gene expression changes in flue-cured tobacco varieties differing in drought tolerance under long-term drought stress are scarce. In this study, drought stress responses of two flue-cured tobacco varieties, LJ851 and JX6007, were comparatively studied at the physiological and transcriptional levels. After exposing to progressive drought stress, the drought-tolerant LJ851 showed less growth inhibition and chlorophyll reduction than the drought-sensitive JX6007. Moreover, higher antioxidant enzyme activities and lower levels of H_2_O_2_, Malondialdehyde (MDA), and electrolyte leakage after drought stress were found in LJ851 when compared with JX6007. Further analysis showed that LJ851 plants had much less reductions than the JX6007 in the net photosynthesis rate and stomatal conductance during drought stress; indicating that LJ851 had better photosynthetic performance than JX6007 during drought. In addition, transcriptional expression analysis revealed that LJ851 exhibited significantly increased transcripts of several categories of drought-responsive genes in leaves and roots under drought conditions. Together, these results indicated that LJ851 was more drought-tolerant than JX6007 as evidenced by better photosynthetic performance, more powerful antioxidant system, and higher expression of stress defense genes during drought stress. This study will be valuable for the development of novel flue-cured tobacco varieties with improved drought tolerance by exploitation of natural genetic variations in the future.

## Introduction

Drought is a major environmental stress factor influencing crop growth, development and yield (Boyer, 1982; Luo, 2010). It is estimated that arid and semi-arid regions account for about 30% of total inter-tilled cropland worldwide (Sivakumar et al., 2005). Thus, water deficit has become a severe threat to sustainable agriculture (Castroluna et al., 2014). Drought stress often leads to a series of morphological variations, physiological and biochemical changes, and gene expression regulation (Shinozaki and Yamaguchi-Shinozaki, 2007). When subjected to drought stress, plants respond and adapt to the adverse conditions by triggering antioxidant defense system to maintain cellular function (Hasegawa et al., 2000; Kawasaki et al., 2001; Zhu, 2002).

Tobacco is an important economic crop with the leaf representing the primary product, and its productivity is vulnerable to drought. In flue-cured tobacco production, drought stress affects tobacco growth at the rosette, vigorous growth, flowering, and maturing stages (Wang et al., 1996; Shang et al., 2010). Moreover, drought occurred at the vigorous growing stage has the most impact on yield and quality of tobacco leaf. During this stage, drought stress resulted in decreases of the biomass and reducing sugar content, and increases of total nitrogen and nicotine contents in flue-cured tobacco (Wu, 1998). Thus, drought is becoming a very important limiting factor for flue-cured tobacco production in the world. To solve this problem, it is the key to developing and popularizing drought-resistant flue-cured tobacco varieties. For this aim, a basic understanding of physiological and molecular responses of tobacco plants to drought stress is essential.

Previous studies have focused on disease resistance, abiotic stress tolerance, and secondary metabolites in transgenic tobacco plants in recent years (Charity et al., 2005; Yang et al., 2007, 2016; Oh et al., 2012). However, comprehensive and comparative analyses of physiological, biochemical and gene transcripts changes in flue-cured varieties differing in drought tolerance under long-term drought stress are scarce. In our lab, we have found that LJ851 had a good performance after 2-week-drought treatment, whereas JX6007 showed severe damage after the stress by screening dozens of flue-cured tobacco varieties under drought conditions at the vigorous growing stage. Here, we hypothesized that there might be differential responses to drought stress in both flue-cured tobacco varieties. To test this idea, we exposed LJ851 and JX6007 plants to drought stress for 2 weeks and investigated their differential responses at physiological, biochemical and gene expression levels under progressive drought conditions. This study may be helpful for developing novel drought-resistant flue-cured tobacco varieties.

## Materials and methods

### Plant material and drought treatment

*Nicotiana tabacum* cv. LJ851 (drought-tolerant) and JX6007 (drought-sensitive) were used in this study. LJ851 was developed from Heilongjiang Institute of Tobacco Science and JX6007 was developed from Shandong Institute of Tobacco Science. Tobacco seeds were surface disinfected in 75% ethanol for 3 min and in 5% sodium hypochlorite for 15 min. The germinated seeds were transferred to plastic pots (22 cm diameter at top, 13 cm diameter at bottom, and 17 cm height) filled with equal quantity pre-autoclaved vermiculite. The plants were grown at approximately 26°C with 60% relative humidity, a photoperiod of 16/8 h (day/night) and light intensity of 300 μmol m^−2^ s^−1^ in the greenhouse.

Drought stress was given to 5-week-old potted plants by withholding water in the soil for 14 days (the soil moisture content was progressively reduced to around 18%). This treatment, in pilot experiments, had been shown to constitute significant stress (about 80% of the drought-sensitive plants had seriously wilted). Soil moisture was measured daily using a Soil Moisture Meter (ECA-SW1, TuoPu Bio Co., Qingdao, China). The control pots were irrigated every 3 days. The experimental design was a randomized complete block with two treatments (LJ851 and JX6007 well-watered, and water stress) arranged in individual pots with 15 plants per treatment (five plants per pot) and three replicates each. Upon 6 (about 60% soil moisture), 10 (about 30% soil moisture), and 14 days (about 18% soil moisture) of drought treatment, leaf or root samples from control (about 85% soil moisture) or treated plants were collected and frozen immediately in liquid N_2_, and kept at −80°C until use.

### Determination of MDA, Ion leakage and H_2_O_2_ content

Malondialdehyde (MDA) content was determined as described by us and others (Draper and Hadley, 1990; Liu et al., 2014). Leaves were homogenized in 5% (w/v) trichloroacetic acid (TCA) and reacted with an equal volume of 0.67% (w/v) thiobarbituric acid (TBA) in a boiling water bath for 30 min. After cooling, the mixture was centrifuged and the supernatant was used to measure the absorbance at 532 nm and corrected for nonspecific turbidity by subtracting the absorbance at 600 and 450 nm.

Ion leakage (IL) was measured and calculated according to the method described by us (Huo et al., 2016). Tobacco leaves were cut into strips and incubated in distilled water for 12 h. The initial conductivity (C1) was measured with a conductivity meter (IS228, Shanghai, China). The samples were then boiled for 30 min to result in complete IL. After cooling down, the electrolyte conductivity (C2) was measured. Electrolyte leakage (C) was calculated according to the equation C (%) = C1/C2 × 100.

H_2_O_2_ content was assayed using the method reported by us (Xia et al., 2012). Frozen leaf samples were homogenized and centrifuged, and the supernatant was collected and reacted with TiCl_4_ and NH_4_OH. After the second centrifuge, the supernatant was discarded and precipitate was washed repeatedly with cold acetone until it turned colorless. The washed precipitate was dissolved in 20 ml 2 M H_2_SO_4_, and then measured absorbance at 415 nm against a blank. Standard H_2_O_2_ were also treated with TiCl_4_ and subjected to the same procedure.

### Determination of chlorophyll and relative leaf water contents

Relative leaf water content (RLWC) was measured as described previously by Quan et al. (2016). Leaf samples at the same part of the plants were harvested at 6, 10, and 14 days under control and drought conditions, respectively. The RLWC was calculated according to the formula: RLWC = (FW − DW)/FW × 100%, where FW is the leaf fresh weight and DW is the dry weight (Quan et al., 2016).

Total chlorophyll content was determined as described by Arnon (1949). Leaf samples were ground in 80% acetone and the homogenate was centrifuged. Then, absorbance of the supernatant was measured at 645 and 663 nm using a spectrop-hotometer (Hitachi U2000, Japan).

### Determination of leaf water potential

Leaf water potential (LWP) was measured as described previously (Cho and Hong, 2006). Leaves of the same position and size from both tobacco varieties were sampled at 6, 10, and 14 days under control and drought conditions, respectively. (LWP) was measured using a C-52 thermocouple sample chamber connected to a Dew point micro-voltmeter (HR-33T, WESCOR, USA).

### Measurement of SOD, CAT, and POD activities

Activities of SOD, CAT, and POD were spectro-photometrically measured according to others and our previous methods (Ahmedi et al., 2009; Liu et al., 2014). Total SOD activity was assayed by monitoring the inhibition of photochemical reduction of nitro blue tetrazolium (NBT). CAT activity was determined by following the consumption of H_2_O_2_ at 240 nm. POD activity was determined according to the methods of Huo et al. (2016). Protein concentration was determined as described by Bradford (1976).

### Measurement of net photosynthetic rate, stomatal conductance and intercellular CO_2_ concentration

The net photosynthetic rate (Pn), stomatal conductance (Gs), and intercellular CO_2_ concentration (Ci) were determined using the LI-6400 portable photosynthesis analyzer (LI-COR, Lincoln, NE, USA) as described by us (Huo et al., 2016). These parameters were measured under the following conditions: 800 μmol m^−2^ s^−1^ photosynthetic photon flux density, 500 μmol s^−1^ flow rate, leaf temperature 30 ± 2°C, and relative humidity 60 ± 1%. Before treatment, the leaves were illuminated for more than 1 h to maintain stomatal opening. In each assay, the second leaf beneath the top leaf was measured on each plant, and four measurements per leaf were recorded.

### Measurement of nicotine and reducing sugars contents

Leaf samples (1.0 g) from control and stressed plants were collected, frozen in liquid N2, and lyophilized. Alkaloids were extracted from dried samples and measured by gas chromatography-mass spectrometry (GC/MS) using a split sampling mode as described in Goossens et al. (2003). Nicotine (Sigma Aldrich) was used as the internal standard.

Concentration of reducing sugars was determined as described by Somogy (1952). 0.5 g of leaf samples was pulverized with distilled water and heated to reach boiling point. The crude extracts were filtrated and transferred to test tubes containing copper sulfate solution. The tubes were incubated for 20 min in water bath 100°C. When the tubes were cooled, phosphomolybdic acid was added and blue color appeared. The test tubes were thoroughly agitated until the color was evenly distributed in the tube. Absorbance was determined in 600 nm by spectrophotometer and concentration of the reducing sugars was calculated by drawing standard curve. The results were calculated and presented as mg per g of fresh weight. In these assays, experiments were repeated three times, which produced similar results.

### RNA isolation, quantification, and cDNA synthesis

Total RNA was isolated using Plant Total RNA kit (Invitrogen, USA). RNA quality and quantity were determined using a Nanodrop 2,000 spectrophotometer (Thermo Scientific, USA). Only RNA samples with A260/A280 between 1.8 and 2.1 and A260/A230 between 2.0 and 2.2 were used. Total RNA integrity was checked via 1.2% agarose gel electrophoresis under denaturing conditions. RNA samples were treated with RQ1 RNase-Free DNase (Promega, USA). cDNA was synthesized from 2 μg of total RNA using the SuperScript^TM^ RT kit (Thermo Scientific, USA) in a 20 μL- reaction volume according to the manufacturer's recommendations. cDNA was stored at −20°C until further use.

### Quantitative real-time PCR

The quantitative real-time PCR (qPCR) was performed in 96-well white plates using an IQ5 Real Time PCR (Bio-Rad, Hercules, CA) with three biological replicates and three technical replicates. The 20 μL reaction mixture contained 1 μL cDNA diluted 20-fold, 0.5 μM of each gene-specific primer (Table S1) and 10 μL master mix (SYBR Green Supermix, Thermo Scientific, USA). The cycling condition is as follows: pre-denaturation, 95°C, 3 min; then 40 cycles at 94°C 10 s (denaturation), 58°C 20 s (annealing), 72°C 20 s (extension), followed by a melting curve analysis to confirm the correct amplification of target gene fragments and the lack of primer dimmers. The PCR products were also run on 2% agarose gels to make sure the specificity of the expected PCR product. PCRs with each primer pair were also performed on samples lacking cDNA template (no template controls). The amplification efficiency of the genes were assessed according to the method as described by Coito et al. (2012). All cDNA samples were diluted 20 fold and were amplified in duplicate in two independent PCR runs. The tobacco *Actin2* and *Tubulin* were used as reference genes (Xia et al., 2013) and the transcript levels of genes were calculated according to the 2^−ΔΔCt^ method (Livaka and Schmittgen, 2001). All qPCR experiments followed the MIQE guidelines (Bustin et al., 2009).

### Statistical analysis

The data are presented as the mean ± SE of three replicates and were analyzed by a simple variance analysis (ANOVA). The differences between the means were compared by Student's *t*-test at *P* < 0.05. For principal component analysis (PCA), the data of the physiological and gene expression parameters were converted into the drought stress index (DSI). DSI was calculated using the formula: DSI = (value of trait under stress condition)/(value of trait under controlled condition) × 100 (Wójcik-Jagła et al., 2013). SPSS statistical software (version 17.0) was used to perform PCA of the parameters.

## Results

### Effects of drought stress on plant growth and leaf water status of two tobacco varieties

**Figure 1:**
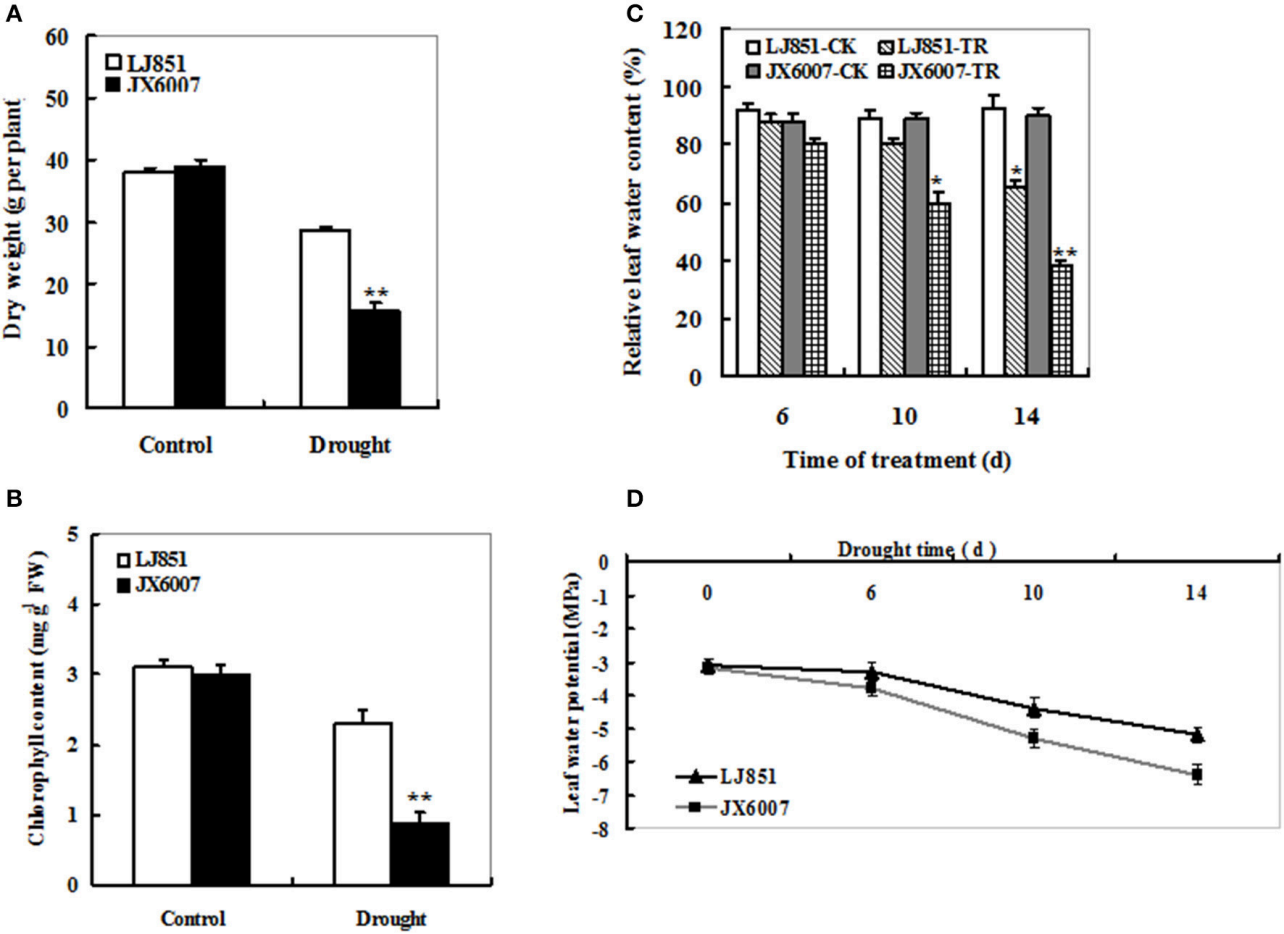
**Effects of drought stress on dry weight (A)**, chlorophyll content **(B)**, relative leaf water content **(C)** and leaf water potential **(D)** in both flue-cured tobacco varieties. LJ851-CK stands for LJ851 under control conditions; LJ851-TR stands for LJ851 under drought conditions; JX6007-CK stands for JX6007 under control conditions; JX6007-TR stands for JX6007 under drought conditions. Bar indicates SE. ^*^t-test, with *P* < 0.05; ^**^t-test, with *P* < 0.01.

To analyze the difference of drought tolerance between two tobacco varieties, 5-week-old plants were subjected to well-watered and drought conditions, respectively. After 14 days, both types of tobacco plants had no significant differences in plant biomass (Figure 1A) and chlorophyll content (Figure 1B) under well-watered conditions. In contrast, under drought stress conditions, almost all of the JX6007 plants had seriously wilted while a few leaves of the LJ851 plants had begun to curl. Accordingly, compared with their corresponding controls, significant decreases in plant dry weight and chlorophyll content were observed in the JX6007 (59 and 70% reductions, respectively) under water stress conditions (Figures 1A,B). Then, we measured the leaf water content (LWC) under control and drought conditions. As shown in Figure 1C, the two varieties showed gradually decreased LWC when subjected to drought stress. Upon 10 days of the drought stress, the LWC had significant reduction in the sensitive variety JX6007, but not in the J851. After 14 day-drought stress, both varieties showed significant reductions in the LWC (Figure 1C). Finally, dynamic changes in (LWP) in both varieties were examined during progressive drought stress (Figure 1D). As shown in Figure 1D, the LWP showed clear decreases in both types of plants during 14 days of the stress, but the magnitudes of decrease were differential. This can be seen upon the 10 and 14 days of drought, in which JX6007 plants showed bigger decreases in the LWP than LJ851 (Figure 1D). Together, LJ851 suffered less harmful effects by drought stress on plant growth and leaf water status compared with JX6007.

### Changes of MDA, Ion leakage and H_2_O_2_ accumulation in two tobacco varieties under progressive drought stress

**Figure 2:**
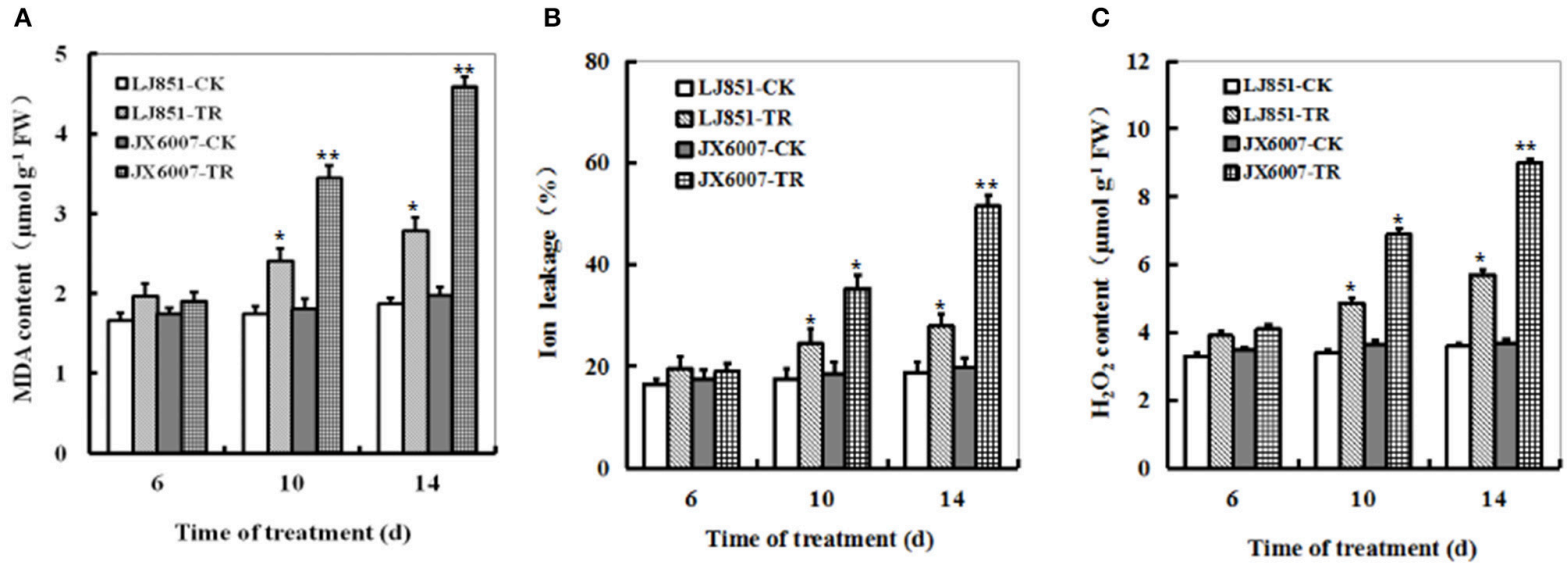
**Dynamic changes of MDA content (A)**, Ion leakage **(B)**, and H_2_O_2_ **(C)** in both genotypes of flue-cured tobacco plants during 14 days of drought stress. LJ851-CK stands for LJ851 under control conditions; LJ851-TR stands for LJ851 under drought conditions; JX6007-CK stands for JX6007 under control conditions; JX6007-TR stands for JX6007 under drought conditions. Bar indicates SE. ^*^t-test, with *P* < 0.05; ^**^t-test, with *P* < 0.01.

Contrasting drought tolerance between LJ851 and JX6007 prompted us to detect the difference in lipid peroxidation. MDA, a product of lipid peroxidation was measured between LJ851 and JX6007 plants during 14 days of drought stress. Under drought conditions, compared with corresponding controls, MDA levels showed clear increases between two types of plants, but the magnitudes of increase were differential. After 14 days of drought, the MDA content was significantly higher in JX6007 (132% increase) than in LJ851 (only 49% increase), suggesting that the LJ851 plants suffered less membrane damage than the JX6007 (Figure 2A). IL measurement also showed that the LJ851 plants had less IL than the JX6007 upon 10 and 14 days after drought stress (Figure 2B), further suggesting that LJ851 plants were subjected to less membrane injury.

The dynamic changes of H_2_O_2_ accumulation levels were examined in the LJ851 and JX6007 plants during 14 days of drought stress. upon 6 days after drought, no significant difference in H_2_O_2_ levels was observed in both varieties between stress and control conditions (Figure 2C). After 6 days, compared with corresponding controls, H_2_O_2_ levels showed significant increases between both types of plants, but the magnitudes of increase were differential. This can be seen upon the 14 days of drought, in which JX6007 plants showed a 143% increase, while LJ851 plants only exhibited a 58% increase (Figure 2C). No significant differences in MDA, H_2_O_2_ or IL were observed in both varieties under control conditions (Figures 2A–C). These physiological indices demonstrated that lower ROS accumulation and lipid peroxidation in LJ851 may be correlated to its higher tolerance to drought stress.

### Changes of antioxidant enzyme activities in two tobacco varieties under progressive drought stress

**Figure 3:**
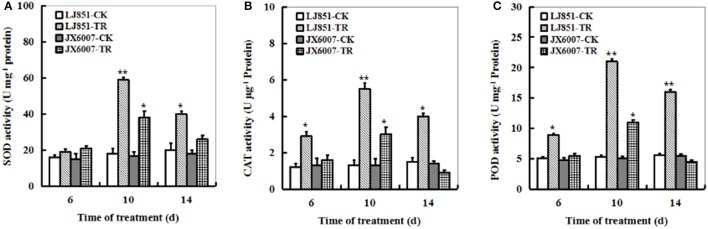
**Dynamic changes of SOD (A)**, CAT **(B)**, and POD **(C)** activities in both types of tobacco plants during 14 days of drought stress. LJ851-CK stands for LJ851 under control conditions; LJ851-TR stands for LJ851 under drought conditions; JX6007-CK stands for JX6007 under control conditions; JX6007-TR stands for JX6007 under drought conditions. Bar indicates SE. ^*^t-test, with *P* < 0.05; ^**^t-test, with *P* < 0.01.

The dynamic changes of three significant antioxidant enzymes SOD, CAT and POD activities were examined in both types of plants during 14 days of drought stress. The LJ851 plants showed much higher SOD, CAT, and POD activities than JX6007 plants under drought conditions, and the highest was observed upon 10 days after the stress treatment (Figures 3A–C). The activities of CAT and POD increased suddenly in LJ851 plants when they were exposed to drought upon 6 days, and then maintained higher levels during drought stress. Unlike CAT and POD, the activity of SOD in LJ851 plants significantly increased 10 days after stress. Noticeably, activities of the three enzymes in the LJ851 maintained higher levels till 14 days after drought, but not in the JX6007 (Figures 3A–C). These results imply that higher levels of antioxidant enzyme activities in LJ851 might help to scavenge toxic levels of ROS induced by drought stress, and to show higher drought tolerance.

### Changes of nicotine and reducing sugars in two tobacco varieties under progressive drought stress

**Figure 4:**
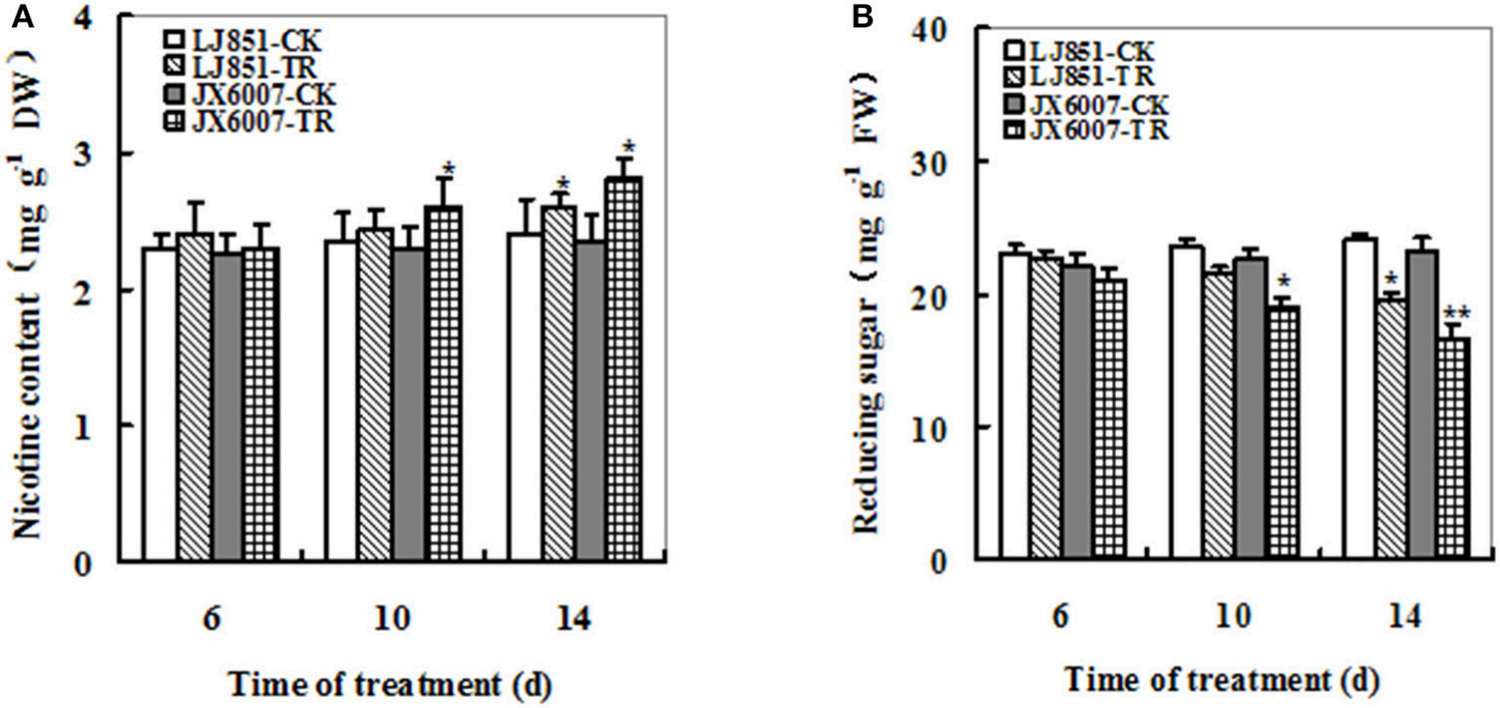
**Dynamic changes of Nicotine (A)** and Reducing sugar **(B)** contents in both types of flue-cured tobacco plants during 14 days of drought stress. LJ851-CK stands for LJ851 under control conditions; LJ851-TR stands for LJ851 under drought conditions; JX6007-CK stands for JX6007 under control conditions; JX6007-TR stands for JX6007 under drought conditions. Bar indicates SE. ^*^t-test, with *P* < 0.05; ^**^t-test, with *P* < 0.01.

Nicotine and reducing sugars can affect taste of smoking and are important parameters for tobacco leaf quality. Under well-watered conditions, no significant difference was observed in nicotine content of two varieties. After drought stress, nicotine content showed an obvious increase in both varieties; moreover, JX6007 exhibited significantly higher nicotine content than LJ851 upon 10 and 14 days of drought treatment (Figure 4A). Dynamic changes in reducing sugars in both varieties were also measured during drought stress. The content of reducing sugars displayed a great decrease when subjected to drought stress in two varieties and the LJ851 revealed more reducing sugars than JX6007 upon 10 and 14 days of the stress (Figure 4B). Together, the higher nicotine and lower reducing sugars in JX6007 might imply that tobacco leaf quality in JX6007 was susceptible to be deteriorated by drought.

### Changes of photosynthetic parameters and transcription of photosynthesis-related genes in two tobacco varieties under drought stress

**Figure 5:**
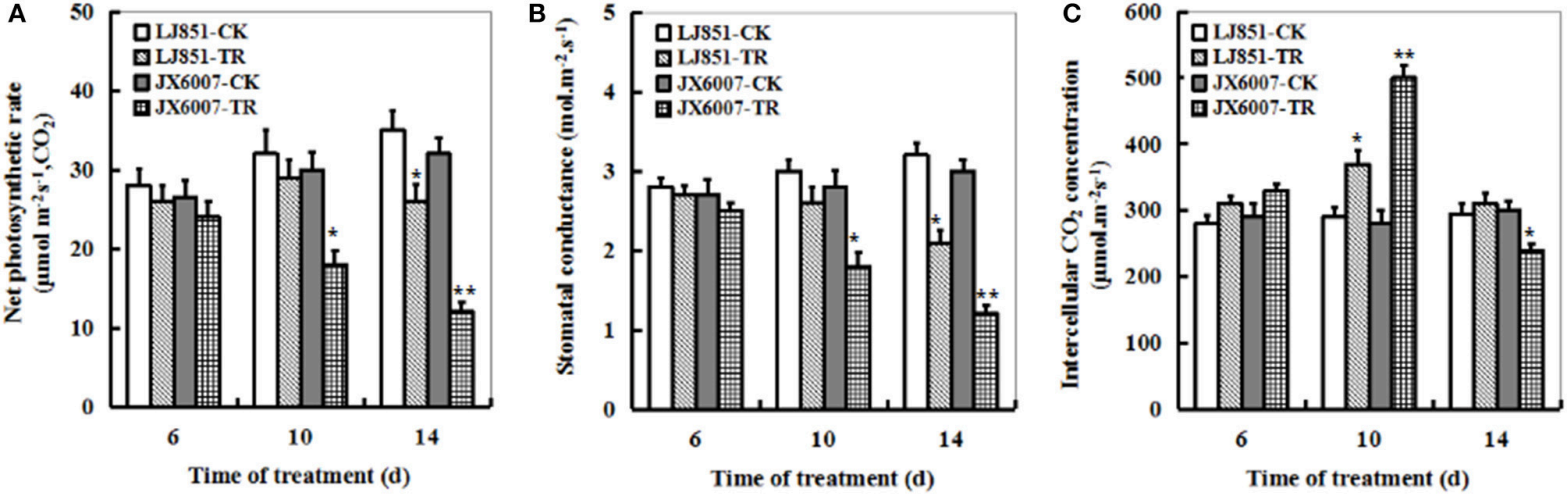
**Dynamic changes of photosynthetic parameters in both types of tobacco plants during 14 days of drought stress. (A)** Net photosynthetic rate; **(B)** Stomatal conductance; **(C)** Intercellular CO_2_ concentration. LJ851-CK stands for LJ851 under control conditions; LJ851-TR stands for LJ851 under drought conditions; JX6007-CK stands for JX6007 under control conditions; JX6007-TR stands for JX6007 under drought conditions. Data are means ± SE calculated from three replicates. Bar indicates SE. ^*^t-test, with *P* < 0.05; ^**^t-test, with *P* < 0.01.

The dynamic changes of the foliar photosynthetic gas exchange parameters during 14 days of drought stress were measured in both varieties as shown in Figure 5. Upon 6 days of the stress, there was no significant difference in leaf net photosynthetic rate (*Pn*) between LJ851 and JX6007 (Figure 5A). Upon 10-d or 14-d of drought stress, however, the decreases in JX6007 were greater (40 and 62% reductions for 10- and 14-d, respectively) than those observed in LJ851 (9 and 25% reductions for 10- and 14-d, respectively). Relative changes in stomatal conductance (*Gs*) were similar to the changes seen in *Pn* after drought stress, with the same trend as in *Pn* but to a smaller extent (Figure 5B). However, upon the 10-d drought stress, JX6007 plants were higher than LJ851 in intercellular CO_2_ concentration (*Ci*); showing an inverse relationship to *Gs* and *Pn* (Figure 5C).

**Figure 6:**
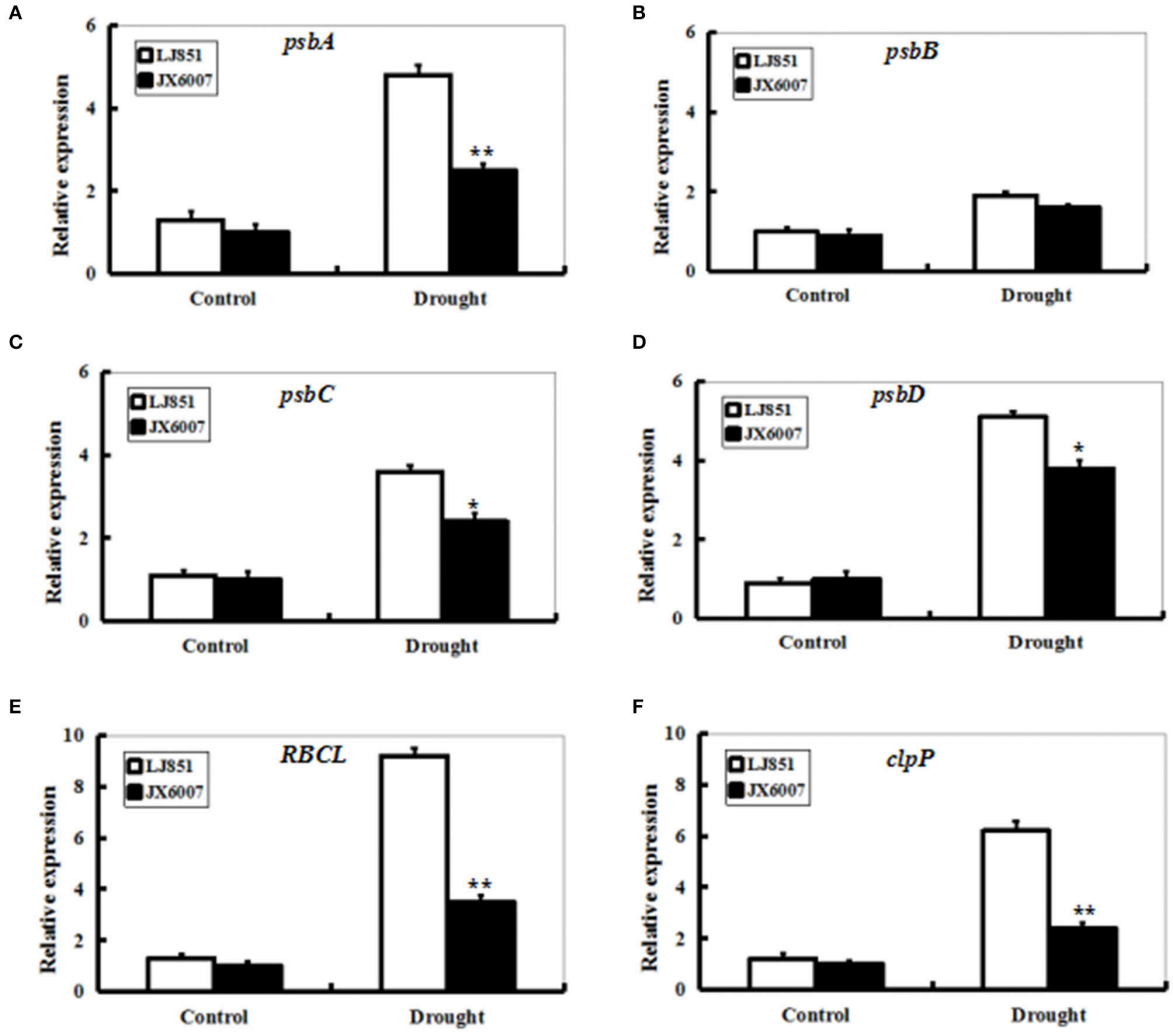
**Expression profiles of genes for photosynthesis-related proteins in both types of tobacco plants after drought stress**. RNA was extracted from stressed leaves sampled after 10 days of drought treatment and reverse-transcribed to synthesize cDNA, which was used for qPCR analysis with primers specific for six photosynthesis-related genes *psbA* **(A)**, *psbB* **(B)**, *psbC* **(C)**, *psbD* **(D)**, *RBCL* **(E)**, and *clpP1***(F)**. Data represented means ± SE of three biological replicates. Bar indicates SE. ^**^t-test, with *P* < 0.01; ^*^t-test, with *P* < 0.05.

Transcript levels of photosynthesis II-related genes *psbA, psbB, psbC, psbD, RBCL, and ClpP1* were detected by qPCR between LJ851 and JX6007 plants after 10-d drought stress (Figure 6). Under control conditions, transcript levels of these six genes underwent no significant changes. After 10 days of drought treatment, except for the gene *psbB*, transcript levels of all the genes significantly increased in both types of plants. In particular, mRNA levels of the five genes in LJ851 plants were significantly higher than those in JX6007 (Figure 6). This demonstrates that chloroplast-encoded gene transcripts in LJ851 plants had more increases than those in JX6007 during drought stress. These results indicate that LJ851 plants showed more powerful photosynthetic performance during drought stress.

### Transcriptional changes of several categories of drought-responsive genes in two tobacco varieties under drought stress

**Figure 7:**
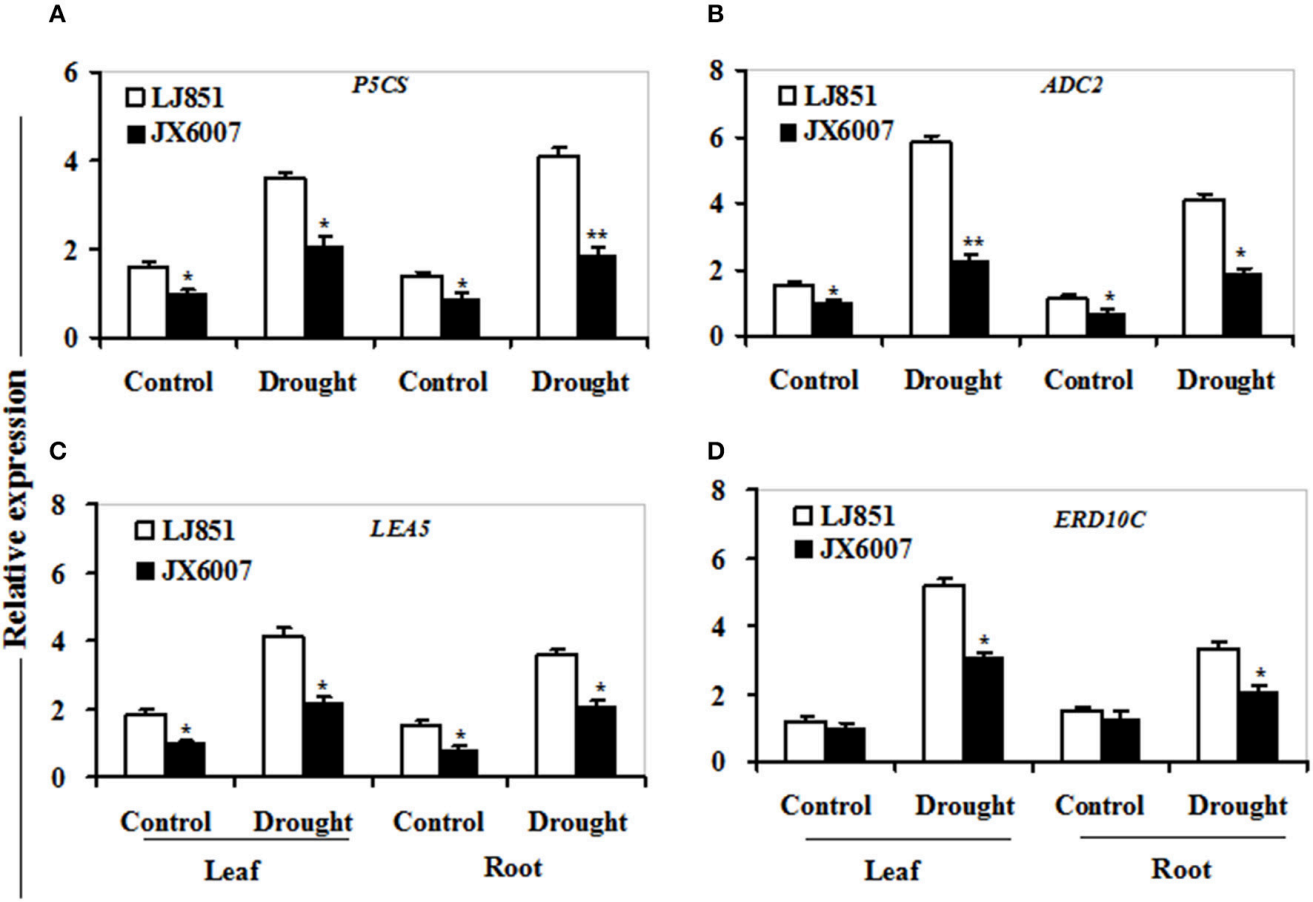
**Transcriptional expression of osmolyte biosynthesis and dehudrin-type genes in leaf and root under drought stress**. RNA was extracted from 10-day stressed leaves and roots and reverse-transcribed to synthesize cDNA, which was used for qPCR analysis with primers specific for four genes *P5CS* **(A)**, *ADC2* **(B)**, *LEA5* **(C)**, and *ERD10C* **(D)**. Data represented means ± SE of three biological replicates. Bar indicates SE. ^**^t-test, with *P* < 0.01; ^*^t-test, with *P* < 0.05.

**Figure 8:**
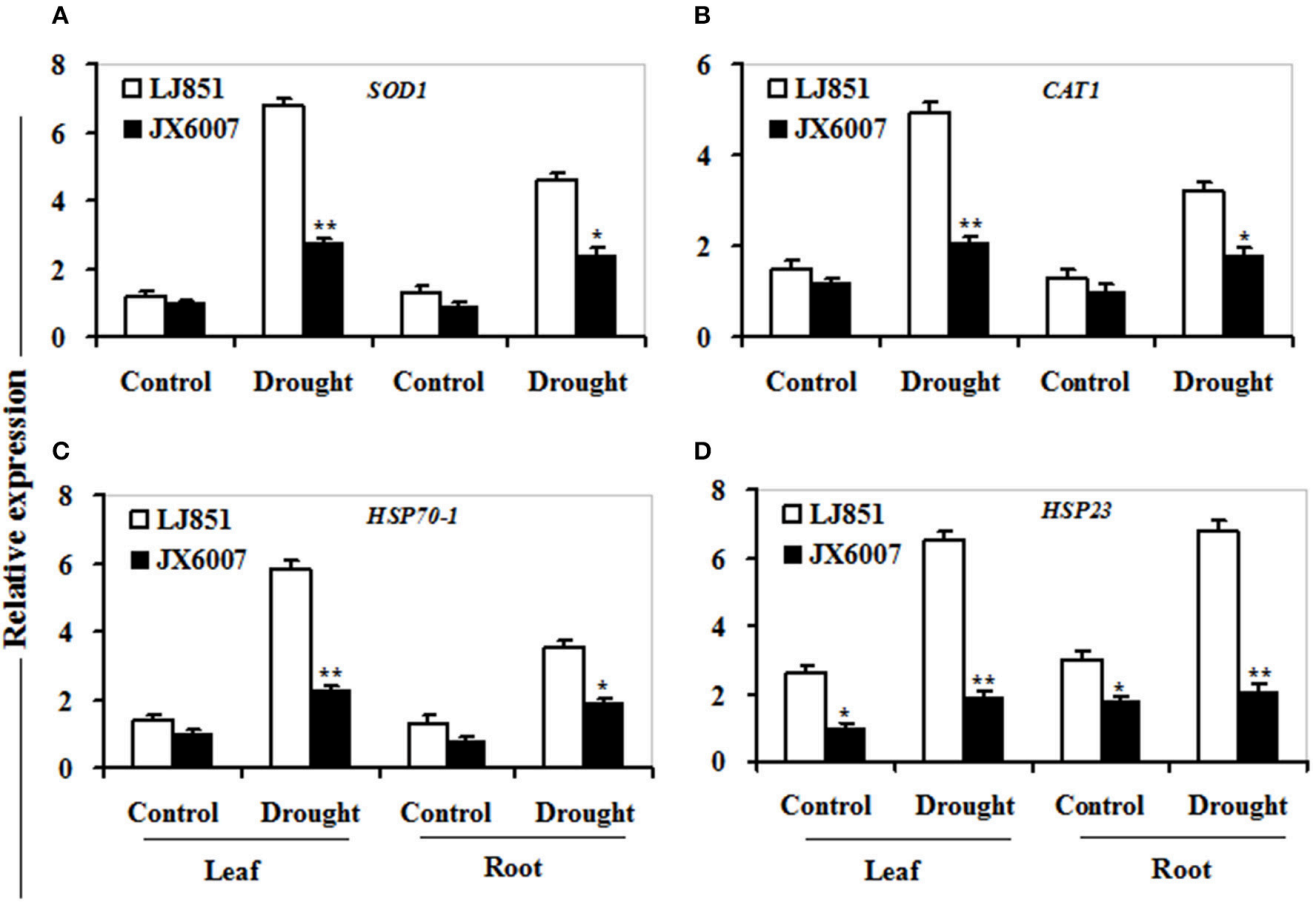
**Transcriptional expression of antioxidant-related and chaperon proteins in leaf and root under drought stress**. RNA was extracted from 10-day stressed leaves and roots and reverse-transcribed to synthesize cDNA, which was used for qPCR analysis with primers specific for four genes *SOD1* **(A)**, *CAT1* **(B)**, *HSP70-1* **(C)**, and *HSP23* **(D)**. Data represented means ± SE of three biological replicates. Bar indicates SE. ^**^t-test, with *P* < 0.01; ^*^t-test, with *P* < 0.05.

**Figure 9:**
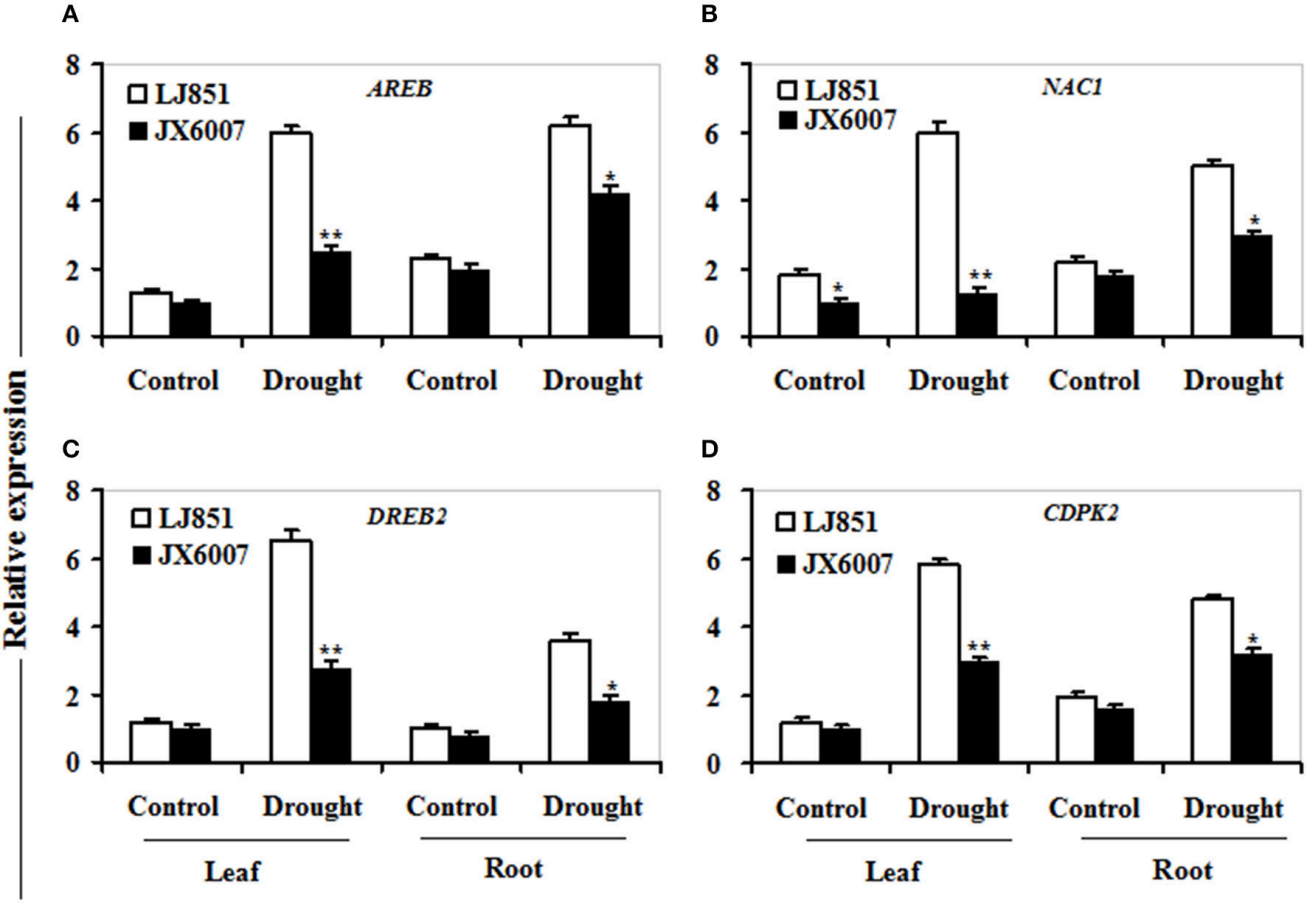
**Transcriptional expression of signaling and transcription factor genes in leaf and root under drought stress**. RNA was extracted from 10-day stressed leaves and roots and reverse-transcribed to synthesize cDNA, which was used for qPCR analysis with primers specific for four genes *AREB* **(A)**, *NAC1* **(B)**, *DREB2* **(C)**, and *CDPK2* **(D)**. Data represented means ± SE of three biological replicates. Bar indicates SE. ^**^t-test, with *P* < 0.01; ^*^t-test, with *P* < 0.05.

It has been reported that numerous genes are regulated by drought stress, including osmolyte biosynthesis genes, defense and antioxidant-related genes, dehydrin-type genes, chaperons, signaling, and transcription regulation-related genes (Zhu, 2002; Ranjan et al., 2012; Vishwakarma et al., 2017). In this study, we analyzed transcriptional changes of five categories of drought-responsive genes including 12 genes in leaves and roots of the two contrasting tobacco varieties by qPCR upon 10 days after drought stress (Figures 7–**9**).

As shown in Figure 7, transcriptional expression of two osmolyte biosynthesis genes *P5CS* and *ADC2*, and two dehydrin-type genes *LEA5* and *ERD10C* in both varieties were examined. Under control conditions, the transcripts of all the genes in both varieties showed no significant differences whatever in leaves or roots, except for *ERD10C* (Figure 7). After drought stress, expression of the four genes was changed and induced by water deficiency in both varieties. Moreover, the expression levels of these genes in LJ851 were obviously higher than those in JX6007 either in leaves or roots (Figure 7). Subsequently, transcriptional levels of two antioxidant-related genes *SOD1 and CAT1*, and two chaperon-encoded genes *HSP70-1* and *HSP23* in both varieties were detected. Under control conditions, the transcripts of these genes in both varieties showed no significant differences whatever in leaves or in roots (Figure 8). After drought stress, the expression of these genes was significantly induced by drought (Figure 8). Moreover, the expression levels of these four genes in the tolerant variety LJ851 were significantly higher than those in JX6007 in both leaves and roots (Figure 8). Finally, the transcriptional expression of signaling (*CDPK2*) and transcription-related genes (*AREB, NAC1*, and *DREB2*) in leaves and roots under drought stress were examined. Compared to their controls these four genes showed high transcript levels after drought stress in both varieties whatever in leaves or roots (Figure 9). Noticeably, the expression levels of the *AREB* in LJ851 were quite high in both leaves and roots under drought conditions (Figure 9A). These data indicate that LJ851 showed better drought tolerance than JX6007 partially due to differential expression of these stress-responsive genes during drought stress.

### Quantitative relations between physiological, biochemical parameters, and gene transcript levels by PCA

**Figure 10:**
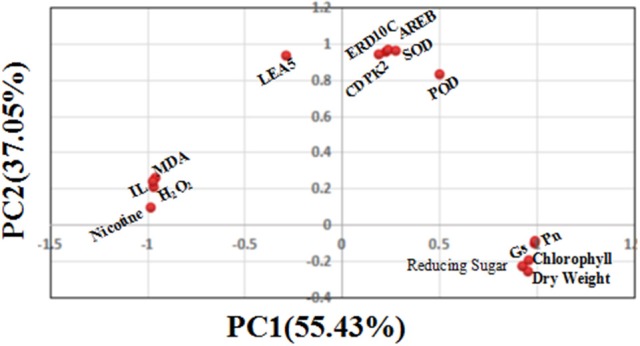
**Scatter plot of the top two principal components (including 15 physiological biochemical and gene transcript parameters) under drought stress**. The proportion of variance for principal component analysis based on the drought stress index (DSI) of 15 physiological traits is shown in the figure, showing that PC1 and PC2 explain 92.48% of total variation.

To further understand the quantitative relations between physiological, biochemical and gene transcript parameters, PCA using the DSI values of 15 physiological, biochemical and molecular measurements was performed to determine their contributions to drought tolerance. As shown in Figure 10, the first principal component (PC1) explained approximately 55.43% of the variance, which mainly included biomass factor (dry weight per plant), oxidation substances (MDA, H_2_O_2_, and IL), metabolites (nicotine and reducing sugars) and photosynthetic factors (Pn, Gs, and chlorophyll content). The second principal component (PC2) explained 37.05% of the variance, which included antioxidant enzymes (SOD and POD) and drought defense genes (*AREB, CDPK2, LEA5*, and *ERD10C*). Together, the two components (PC1 and PC2) could explain 92.48% of total variance. This suggested that there exists the quantitative relations between physiological, biochemical and gene transcript parameters as established by PCA, and both principal components including 15 parameters for assessing drought tolerance in the flue-cured tobacco are reliable in this study.

## Discussion

Drought is a major stress factor reducing agricultural productivity. Flue-cured tobacco is one of the most important sources of tobacco leaf raw materials. Therefore, it is important to identify superior drought-tolerant flue-cured tobacco varieties and to better understand their responses to drought at multiple levels. LJ851 and JX6007 were two commonly planted flue-cured tobacco varieties in most regions of China, but differed in drought stress responses. No study has been conducted to compare the physiological and molecular responses of both genotypes to the progressive drought stress yet. In this work, we found that LJ851 and JX6007 differed in their responses to drought stress at physiological and gene transcriptional levels.

### LJ851 had better photosynthetic performance than JX6007 under drought stress

Photosynthesis is an important metabolic process in higher plants and photosynthetic apparatus is susceptible to be impaired during drought stress (Chaves et al., 2009). Alterations in photosynthetic parameters under drought stress are good indicators of drought tolerance for plants. As shown in Figures 5A–C, the *Pn* and *Gs* of both LJ851 and JX6007 plants decreased upon 10-d drought stress. Particularly, the *Gs* in LJ851 plants has less reductions than that in JX6007 after drought (Figure 5B). This change trend in *Gs* is similar to that in *Pn* between LJ851 and JX6007 plants, indicating that the higher *Pn* in LJ851 plants was mainly dependent on *Gs* regulation. However, the changes observed in *Ci* showed an inverse relationship to *Gs* and *Pn* (the *Ci* of JX6007 was higher than LJ851 plants) (Figure 5C). This was partly due to the different decrease of *Pn* in both LJ851 and JX6007 plants (Figure 5A). This could result in higher CO_2_ assimilation and lower *Ci* in the LJ851 plants than JX6007, thus photosynthesis and growth inhibition in the LJ851 were ameliorated under drought stress. This viewpoint was also evidenced by comparisons of the biomass and chlorophyll content between both types of plants, showing that LJ851 had less reductions than JX6007 in both parameters after drought stress (Figures 1A,B). In addition, changes in transcripts of photosynthesis II-related genes between LJ851 and JX6007 plants during drought have demonstrated that chloroplast-encoded gene transcripts in LJ851 plants had more increases than those in JX6007 (Figure 6). These results indicate that LJ851 had better photosynthetic performance than JX6007 under drought stress.

### LJ851 had more powerful antioxidant system and retaining water ability than JX6007 under drought stress

It has been reported that drought would induce oxidative damage, represented as H_2_O_2_ accumulation, lipid peroxidation and electrolyte leakage (Apel and Hirt, 2004; Li et al., 2013; Liu et al., 2014). LJ851 plants showed less H_2_O_2_, MDA and electrolyte leakage than JX6007 under drought stress (Figures 2A–C). Similar differences in H_2_O_2_, MDA and electrolyte leakage between maize genotypes differing in drought tolerance had been observed during drought stress (Chen et al., 2015). Also, Ahmed et al. (2016) found that Tibetan wild barley showed less oxidative damage than cultivated variety under PEG and Al stresses (Ahmed et al., 2016). Furthermore, drought-induced reduction in chlorophyll content is considered to be a typical oxidative stress indicator, which might be attributed to thylakoid membrane damage caused by the increased ROS (Kapanigowda et al., 2013). Thus, chlorophyll content and photosynthetic parameters under drought stress could be used as reliable reference indicators in the selection of drought-adaptive genotypes (Chen et al., 2015). Our results showed that chlorophyll content and photosynthesis activity in LJ851 plants are higher than those in JX6007 plants under drought stress (Figures 1B, 5). In agreement with our results, Fracasso et al. (2016) also found similar differences in chlorophyll content and photosynthetic parameters between *Sorghum bicolor* genotypes with different drought tolerance (Fracasso et al., 2016).

Plants can cope with drought stress effectively by reducing transpirational water loss to keep plants to conserve adequate water status; moreover, leaf water content reflects water status of plants during drought stress (Quan et al., 2016). In this study, LJ851 showed higher leaf water content than the JX6007, suggesting that LJ851 had slower transpirational water loss than JX6007. Noticeably, LJ851 also showed smaller leaf size compared with JX6007 (Data not shown), which contributed to reduce transpiration area under drought stress. These results indicated that LJ851 could maintain higher water status to alleviate cell damage caused by drought stress compared with JX6007. Together, less H_2_O_2_, MDA and electrolyte leakage, and higher chlorophyll content, leaf water content and photosynthesis activity in LJ851 plants indicated that LJ851 suffered less serious injury than the JX6007 under drought stress.

It is well-known that antioxidant enzymes have pivotal roles in protecting plants from ROS-induced oxidative damage (Apel and Hirt, 2004). SOD, POD, and CAT are key enzymes in the active oxygen-scavenging system and increased activities of the enzymes would decrease ROS levels (Apel and Hirt, 2004; Huo et al., 2016). In this study, we detected the activities of antioxidant enzymes between both genotypes of plants during drought stress. Our results showed that higher total SOD, POD, and CAT activities in LJ851 than those in X6007 under drought conditions (Figures 3A–C). Accordingly, lower ROS levels were observed in the LJ851 plants during drought stress (Figure 2C). Consistently, higher antioxidant enzyme activities were also found in the tolerant variety than those in the sensitive one under various environmental stresses, such as salt, drought, heat or high light stress (Jagtap and Bhargava, 1995; Türkan et al., 2005; Khanna-Chopra and Selote, 2007; Wang et al., 2009). These results indicate that LJ851 possessed a more powerful ROS scavenging system than the drought-sensitive JX6007.

### LJ851 had higher transcript levels of stress-responsive genes than JX6007 under drought stress

Drought stress regulates expression of thousands of genes in plants at both transcriptional and post-transcriptional levels (Zhu, 2002). In the present study, transcript levels of 12 stress-responsive genes including osmolyte biosynthesis genes, defense and antioxidant-related genes, dehydrin-type genes, chaperons, signaling and transcription regulation-related genes in LJ851 had much higher increases than those in the drought-sensitive JX6007 during drought stress (Figures 7–9).

The Δ1-pyrroline-5-carboxylate synthetase (P5CS) gene plays a predominant role in drought-induced proline accumulation (Kam and Nam, 2013). As we know, proline acts as an important osmoprotectant and confers abiotic stress tolerance in plants (Ashraf and Foolad, 2007). Remarkably higher expression levels of *P5CS*, especially in roots of LJ851 might indicate greater proline accumulation compared to JX6007 after drought stress (Figure 7A). ADC2 is involved in biosynthesis of polyamines, which function in stress adaptation by acting as osmoticum regulator or membrane stabilizer (Aziz and Larher, 1995; Liu et al., 2007). More drastic induction of these osmolyte biosynthesis genes in the drought-tolerant variety LJ851 (Figures 7A,B) implied that LJ851 plants might synthesize higher levels of proline and polyamines to prevent them from injury and maintain better growth under drought stress (Shi et al., 2010). *LEA5* and *ERD10C* are assumed to play critical roles in combating cellular dehydration (Hundertmark and Kincha, 2008; Kovacs et al., 2008). High transcript levels of these genes in the drought-tolerant variety LJ851 (Figures 7C,D) suggested that LJ851 plants might synthesize more protective proteins for maintaining membrane integrity and cell function to be in better defense against water stress.

Heat shock proteins (HSPs) 70 and 23 are two classes of chaperons, which could protect cells against damage caused by environmental stresses in plants. Overexpression of cytoplasmic *HSP70-1* or mitochondrial *HSP23* contributes to drought stress tolerance in transgenic tobacco (Cho and Hong, 2006; Lee et al., 2012). In this study, drought stress resulted in increased expression of *HSP70-1* and *HSP23*, and LJ851 showed significantly higher *HSP70-1* and *HSP23* expression in both leaves and roots compared to JX6007 under drought stress (Figures 8C,D). The higher levels of *HSP70-1* or *HSP23* in the LJ851 plants might protect cells from drought-induced oxidative damage through chaperons and antioxidant activities.

CDPK2, DREB2, AREB, and NAC1 are important regulatory molecules involved in signal transduction or transcriptional regulation during stress conditions (Shinozaki and Yamaguchi-Shinozaki, 2007; Saibo et al., 2009; Witte et al., 2010). Interestingly, expression patterns of these genes were largely consistent with those functional genes after water stress (Figure 9). Noticeably, compared to other genes, the bZIP transcription factor gene *AREB* in the drought-tolerant LJ851 plants was induced to a higher level than the sensitive JX6007 (Figure 9A); suggesting that abscisic acid signaling might be more active in the drought-tolerant LJ851 than that in the sensitive JX6007 during drought stress. However, it is not clear why the transcripts of these stress-related genes were differential in both genotypes of plants during drought stress.

Taken together, our results showed that LJ851 was more tolerant to drought stress than JX6007 as evidenced by the differences at physiological and transcriptional levels. LJ851 had better photosynthetic performance, which resulted in less reductions in biomass and chlorophyll content during drought stress. In addition, compared to JX6007, higher antioxidant enzymes in LJ851 may contribute to protect cell membrane and photosynthetic machinery from oxidative damage during drought stress. Meanwhile, LJ851 showed significantly higher expression of drought- responsive genes, indicating that LJ851 exhibited better genetic basis against drought stress than JX6007. This study will help to development novel flue-cured tobacco varieties with improved drought tolerance by exploiting natural genetic variations in the future.

## Author contributions

ZX designed the research. XS, FW, YH, and ZX performed research and conducted data analyses. ZX wrote and revised the manuscript.

### Conflict of interest statement

The authors declare that the research was conducted in the absence of any commercial or financial relationships that could be construed as a potential conflict of interest.

## References

[B1] Ahmed I. M. Nadira U. A. Cao F. He X. Zhang G. Wu F. (2016). Physiological and molecular analysis on root growth associated with the tolerance to aluminum and drought individual and combined in Tibetan wild and cultivated barley. Planta 243, 973–985. 10.1007/s00425-015-2442-x 26748913

[B2] Ahmedi C. B. Rouina B. B. Sensoy S. Boukhris M. Abdallah F. B. (2009). Changes in gas exchange, proline accumulation and antioxidative enzyme activities in three olive cultivars under contrasting water availability regimes. Environ. Exp. Bot. 67, 345–352. 10.1016/j.envexpbot.2009.07.006

[B3] Apel K. Hirt H. (2004). Reactive oxygen species: metabolism, oxidative stress, and signal transduction. Annu. Rev. Plant Biol. 55, 373–399. 10.1146/annurev.arplant.55.031903.141701 15377225

[B4] Arnon D. I. (1949). Copper enzymes in isolated chloroplasts: polyphenoloxidase in beta vulgaris. Plant Physiol. 24, 1–15. 10.1104/pp.24.1.1 16654194 PMC437905

[B5] Ashraf M. Foolad M. (2007). Roles of glycine betaine and proline in improving plant abiotic stress resistance. Environ. Exp. Bot. 59, 206–216. 10.1016/j.envexpbot.2005.12.006

[B6] Aziz A. Larher F. (1995). Changes in polyamine titers associated with the proline response and osmotic adjustment of rape leaf discs submitted to osmotic stresses. Plant Sci. 112, 175–186. 10.1016/0168-9452(95)04264-4

[B7] Boyer J. S. (1982). Plant productivity and environment. Science 218, 443–448. 10.1126/science.218.4571.443 17808529

[B8] Bradford M. M. (1976). A rapid and sensitive method for the quantification of microgram quantities of protein utilizing the principal of protein-dye binding. Anal. Biochem. 72, 248–254. 10.1016/0003-2697(76)90527-3 942051

[B9] Bustin S. A. Benes V. Garson J. A. Hellemans J. Huggett J. Kubista M.. (2009). The MIQE guidelines: minimum Information for publication of quantitative real-time PCR experiments. Clin. Chemistry 55, 611–622. 10.1373/clinchem.2008.112797 19246619

[B10] Castroluna A. Ruiz O. M. Quiroga A. M. (2014). Effects of salinity and drought stress on germination, biomass and growth in three varieties of *Medicagosativa L. Avances invest*. Agropec 18, 39–50.

[B11] Charity J. A. Hughes P. Anderson M. A. Bittisnich D. J. Whitecross M. Higgins T. (2005). Pest and disease protection conferred by expression of barley β-hordothionin and Nicotiana alataproteinase inhibitor genes in transgenic tobacco. Funct. Plant Biol. 32, 35–44. 10.1071/FP04105 32689109

[B12] Chaves M. M. Flexas J. Pinheiro C. (2009). Photosynthesis under drought and salt stress: regulation mechanisms from whole plant to cell. Ann. Bot. 103, 551–560. 10.1093/aob/mcn125 18662937 PMC2707345

[B13] Chen D. Wang S. Cao B. Cao D. Leng G. Li H.. (2015). Genotypic variation in growth and physiological response to drought stress and Re-Watering reveals the critical role of recovery in drought adaptation in maize seedlings. Front. Plant Sci. 6:1241. 10.3389/fpls.2015.01241 26793218 PMC4709455

[B14] Cho E. K. Hong C. B. (2006). Over-expression of tobacco NtHSP70-1 contributes to drought-stress tolerance in plants. Plant Cell Rep. 25, 349–358. 10.1007/s00299-005-0093-2 16365678

[B15] Coito J. L. Rocheta M. Carvalho L. Amβncio S. (2012). Microarray-based uncovering reference genes for quantitative real time PCR in grapevine under abiotic stress. BMC Res. Notes 5:220. 10.1186/1756-0500-5-220 22564373 PMC3837474

[B16] Draper H. H. Hadley M. (1990). Malondialdehyde determination as index of lipid peroxidation. Meth. Enzymol. 86, 421–431. 10.1016/0076-6879(90)86135-I 2233309

[B17] Fracasso A. Trindade L. Amaducci S. (2016). Drought tolerance strategies high-lighted by two *Sorghum bicolor* races in a dry-down experiment. J. Plant Physiol. 190, 1–14. 10.1016/j.jplph.2015.10.009 26624226

[B18] Kam G. B. Nam Y. W. (2013). A novel Δ1-pyrroline-5-carboxylate synthetase gene of *Medicago truncatula* plays a predominant role in stress-induced proline accumulation during symbiotic nitrogen fixation. J. Plant Physiol. 170, 291–302. 10.1016/j.jplph.2012.10.004 23158502

[B19] Goossens A. Häkkinen S. T. Laakso I. Seppänen-Laakso T. Biondi S. De Sutter V.. (2003). A functional genomics approach toward the understanding of secondary metabolism in plant cells. Proc. Natl. Acad. Sci. U.S.A. 100, 8595–8600. 10.1073/pnas.1032967100 12826618 PMC166274

[B20] Hasegawa M. Bressan R. Pardo J. M. (2000). The dawn of plant salt tolerance genetics. Trends Plant Sci. 5, 317–319. 10.1016/S1360-1385(00)01692-7 10908874

[B21] Hundertmark M. Kincha D. K. (2008). LEA (Late Embryogenesis Abundant) proteins and their encoding genes in *Arabidopsis thaliana*. BMC Genomics 9:118. 10.1186/1471-2164-9-118 18318901 PMC2292704

[B22] Huo Y. Wang M. Wei Y. Xia Z. (2016). Overexpression of the maize psbA gene enhances drought tolerance through regulating antioxidant system, photosynthetic capability, and stress defense gene expression in tobacco. Front. Plant Sci. 6:1223. 10.3389/fpls.2015.01223 26793207 PMC4709446

[B23] Jagtap V. Bhargava S. (1995). Variation in the antioxidant metabolism of drought tolerant and drought susceptible varieties of *Sorghum bicolor* (L.) Moench. exposed to high light, low water and high temperature stress. J. Plant Physiol. 145, 195–197. 10.1016/S0176-1617(11)81872-9

[B24] Kapanigowda M. H. Perumal R. Djanaguiraman M. Aiken R. M. Tesso T. Prasad P. V.. (2013). Genotypic variation in sorghum [*Sorghum bicolor* (L.) Moench] exotic germplasm collections for drought and disease tolerance. Springerplus 2:650. 10.1186/2193-1801-2-650 24349954 PMC3863401

[B25] Kawasaki S. Borchert C. Deyholos M. Wang H. Brazille S. Kawai K.. (2001). Gene expression profiles during the initial phase of salt stress in rice. Plant Cell 13, 889–905. 10.1105/tpc.13.4.889 11283343 PMC135538

[B26] Khanna-Chopra R. Selote D. S. (2007). Acclimation to drought stress generates oxidative stress tolerance in drought-resistant than susceptible wheat cultivar under field conditions. Environ. Exp. Bot. 60, 276–283. 10.1016/j.envexpbot.2006.11.004

[B27] Kovacs D. Kalmar E. Torok Z. Tompa P. (2008). Chaperone activity of ERD10 and ERD14, two disordered stress-related plant proteins. Plant Physiol. 147, 381–390. 10.1104/pp.108.118208 18359842 PMC2330285

[B28] Lee K. W. Choi G. J. Kim K. Y. Ji H. J. Park H. S. Kim Y. G. (2012). Trangenic expression of MsHsp23 confers enhanced tolerance to abiotic stresses in tall fescue. Asian Australas. J. Anim. Sci. 6, 818–823. 10.5713/ajas.2012.12034 PMC4093096 25049632

[B29] Li Y. Zhang J. Zhang J. Hao L. Hua J. Duan L.. (2013). Expression of an *Arabidopsis molybdenum* cofactor sulphurase gene in soybean enhances drought tolerance and increases yield under field conditions. Plant Biotechnol. J. 11, 747–758. 10.1111/pbi.12066 23581509

[B30] Liu J.-H. Kitashiba H. Wang J. Ban Y. Moriguchi T. (2007). Polyamines and their ability to provide environmental stress tolerance to plants. Plant Biotechnol. 24, 117–126. 10.5511/plantbiotechnology.24.117

[B31] Liu J. Li J. Su X. Xia Z. (2014). Grafting improves drought tolerance by regulating antioxidant enzyme activities and stress-responsive gene expression in tobacco. Environ. Exp. Bot. 107, 173–179. 10.1016/j.envexpbot.2014.06.012

[B32] Livaka K. J. Schmittgen T. D. (2001). Analysis of relative gene expression data using real-time quantitative PCR and the 2^−ΔΔC^T method. Methods 25, 402–408. 10.1006/meth.2001.1262 11846609

[B33] Luo L. J. (2010). Breeding for water-saving and drought-resistance rice (WDR) in China. J. Exp. Bot. 61, 3509–3517. 10.1093/jxb/erq185 20603281

[B34] Oh Y. Baldwin I. T. Gális I. (2012). NaJAZh regulates a subset of defense responses against herbivores and spontaneous leaf necrosis in *Nicotiana attenuata* plants. Plant Physiol. 159, 769–788. 10.1104/pp.112.193771 22496510 PMC3375940

[B35] Quan W. Liu X. Wang H. Chan Z. (2016). Comparative physiological and transcriptional analyses of two contrasting drought tolerant alfalfa varieties. Front. Plant Sci. 6:1256. 10.3389/fpls.2015.01256 26793226 PMC4709457

[B36] Ranjan A. Nigam D. Asif M. H. Singh R. Ranjan S. Mantri S.. (2012). Genome wide expression profiling of two accession of *G.herbaceum L*. in response to drought. BMC Genomics. 13:94. 10.1186/1471-2164-13-94 22424186 PMC3320563

[B37] Saibo N. J. M. Lourenço T. Oliveira M. M. (2009). Transcription factors and regulation of photosynthetic and related metabolism under environmental stresses. Ann. Bot. 103, 609–623. 10.1093/aob/mcn227 19010801 PMC2707349

[B38] Shang X. Liu H. Zhang X. Lin J. Duan W. Yang T. (2010). Growth and physiological characteristics of roots in different flue-cured tobacco varieties under drought stress. Acta Bot. Boreal.-Occident. Sin. 30, 357–361.

[B39] Shi J. Fu X. Peng T. Huang X. Fan Q. Liu J. (2010). Spermine pretreatment confers dehydration tolerance of citrus *in vitro* plants via modulation of antioxidative capacity and stomatal response. Tree Physiol. 30, 914–922. 10.1093/treephys/tpq030 20462936

[B40] Shinozaki K. Yamaguchi-Shinozaki K. (2007). Gene networks involved in drought stress response and tolerance. J. Exp. Bot. 58, 221–227. 10.1093/jxb/erl164 17075077

[B41] Sivakumar M. V. K. Das H. P. Brunini O. (2005). Impacts of present and future climate variability and change of agriculture and forestry in the arid and semi-arid tropics. Clim. Change 70, 31–72. 10.1007/s10584-005-5937-9

[B42] Somogy M. (1952). Notes on sugar determination. J. Biol. Chem. 195, 19–29. 14938350

[B43] Türkan I. Bor M. Özdemir F. Koca H. (2005). Differential responses of lipid peroxidation and antioxidants in the leaves of drought-tolerant *P. acutifolius* Gray and drought-sensitive *P. vulgaris L*. subjected to polyethylene glycol mediated water stress. Plant Sci. 168, 223–231. 10.1016/j.plantsci.2004.07.032

[B44] Vishwakarma K. Upadhyay N. Kumar N. Yadav G. Singh J. Mishra R. K.. (2017). Abscisic acid signaling and abiotic stress tolerance in plants: a review on current knowledge and future prospects. Front. Plant Sci. 8:161. 10.3389/fpls.2017.00161 28265276 PMC5316533

[B45] Wang W. B. Kim Y. H. Lee H. S. Kim K. Y. Deng X. P. Kwak S. S. (2009). Analysis of antioxidant enzyme activity during germination of alfalfa under salt and drought stresses. Plant Physiol. Biochem. 47, 570–577. 10.1016/j.plaphy.2009.02.009 19318268

[B46] Wang Y. Han J. Lin X. (1996). Study on physiological and biochemical responses of flue-cured tobacco to drought stress during early growth of the plants. *Acta Agron*. Sinica 22, 117–121.

[B47] Witte C. P. Keinath N. Dubiella U. Demoulière R. Seal A. Romeis T. (2010). Tobacco calcium-dependent protein kinases are differentially phosphorylated *in vivo* as part of a kinase cascade that regulates stress response. J. Biol. Chem. 285, 9740–9748. 10.1074/jbc.M109.052126 20118232 PMC2843223

[B48] Wójcik-Jagła M. Rapacz M. Tyrka M. Kościelniak J. Crissy K. Żmuda K. (2013). Comparative QTL analysis of early short-time drought tolerance in Polish fodder and malting spring barley. Theor. Appl. Genet. 126, 3021–3034. 10.1007/s00122-013-2190-x 24057106 PMC3838596

[B49] Wu X. (1998). The effects of soil moisture on yield and quality of flue-cured tobacco. Agric. Tech. 4, 3–6.

[B50] Xia Z. Sun K. Wang M. Wu K. Zhang H. Wu J. (2012). Overexpression of a maize sulfite oxidase gene in tobacco enhances tolerance to sulfite stress via sulfite oxidation and CAT-mediated H_2_O_2_ scavenging. PLoS ONE 7:e37383. 10.1371/journal.pone.0037383 22693572 PMC3365070

[B51] Xia Z. Su X. Liu J. Wang M. (2013). The RING-H2 finger gene 1 (RHF1) encodes an E3 ubiquitin ligase and participates in drought stress response in *Nicotiana tabacum*. Genetica 141, 11–21. 10.1007/s10709-013-9702-0 23381133

[B52] Yang L. Li J. Ji J. Li P. Yu L. Abd_Allah E. F.. (2016). High temperature induces expression of tobacco transcription factor NtMYC2a to regulate nicotine and JA biosynthesis. Front. Physiol. 7:465. 10.3389/fphys.2016.00465 27833561 PMC5081390

[B53] Yang X. Xiao Y. Wang X. Pei Y. (2007). Expression of a novel small antimicrobial protein from the seeds of motherwort (*Leonurus japonicus*) confers disease resistance in tobacco. Appl. Environ. Microb. 73, 939–946. 10.1128/AEM.02016-06 17158620 PMC1800757

[B54] Zhu J. K. (2002). Salt and drought stress signal transduction in plants. Annu. Rev. Plant Biol. 53, 247–273. 10.1146/annurev.arplant.53.091401.143329 12221975 PMC3128348

