# Supplementary material for: Comparative Physiological and Molecular Analyses of Two Contrasting Flue-Cured Tobacco Genotypes under Progressive Drought Stress
Source: Front Plant Sci. 2017 May 17;8:827. doi: 10.3389/fpls.2017.00827 (PMC5434153; doi:10.3389/fpls.2017.00827)
Supplement: Supplementary file 1 [file Table1.DOC]

**Supplementary Material**

**Table S1 Primers used in the quantitative real-time PCR assays of this study**

Note: Basic residues are indicated in bold.

| **Primer name** | **Primer sequence** | **Product length/bp** | **Ta/ °C** |
| --- | --- | --- | --- |
| LEA-QF  LEA-QR  ERD10C-QF  ERD10C-QR  CDPK2-QF  CDPK2-QR  AREB-QF  AREB-QR  P5CS-QF  P5CS-QF  ADC2-QF  ADC2-QR  SOD1-QF  SOD1-QR  CAT1-QF  CAT1-QR  HSP70-1-QF  HSP70-1-QR  HSP23-QF  HSP23-QR  DREB-QF  DREB-QR  NAC1-QF  NAC1-QR  PsbA-QF  PsbA-QR  PsbB-QF  PsbB-QR  PsbC-QF  PsbC-QR  PsbD-QF  PsbD-QR  RBCL-QF  RBCL-QR  clpP-QF  clpP-QR  Actin2-QF  Actin2-QR | 5'-TTGTTAGCAGGCGTGGGTAT-3'  5'-CTCTCGCTCTTGTTGGGTTC-3'  5'-ACGGACGAATACGGCAATC-3'  5'-TCTCCTTAATCTTCTCCTTCATCC-3'  5'- AGGTGAGCTTTTCGATAGGATTATT-3'  5'- ACTTCTGGTGCAACATA TAAGGAC-3'  5'-TCTTCACAGCAAAAGCCTCA-3'  5'-GTGACCCCATTATGCAATCC-3'  5'-TGTTGTCATAACCAGTGGCTTCG-3'  5'-GGATTGCCTTTTCATTTGCTTCC-3'  5'-GCCGGCCCTAGGTTGTTGTGTAGATG-3'  5'-AGCGAACAACAAGAGGCAGCTGAAG-3'  5'-GACGGACCTTAGCAACAGG-3'  5'-CTGTAAGTAGTATGCATGTTC-3'  5'-TGGATCTCATACTGGTCTCA-3'  5'-TTCCATTGTTTCAGTCATTCA-3'  5'-GCCTTTATTTAGATCCTTTTGCTC-3'  5'-AATGCGTGAAGATTATGTCGTAGA-3'  5'-AAAGAAACCCCAGATTCCCATAT-3'  5'-GGCAGCCTAAACCTTCTCATAAAC-3'  5'-GGACCCACTTGCTGATTCTT-3'  5'-GCGCCTCCTCATCCATATAA-3'  5'-TTACTCGGTTGAAGAAGCAGAAG-3'  5'-CGACGAAATACTCTAGCCAAATT-3'  5'-AGGACCACCGTTGTATAACCA-3'  5'-GCTGCTCCTCCAGTAGACAT-3'  5'-CGGCGTCAGTTATAGTGATCCTG-3'  5'-GTCCGAAGAAGAAGAGCAAAGCA-3'  5'-GCTTCTCAAGCTCAAGCATTTAC-3'  5'-TTCAACCTACTCAAGTCTAACCCAT-3'  5'-ACATTCCGTGCTTTTAACCCAACT-3'  5'-TCAGGGCTAGACCGACTACTCCA-3'  5'-GACCGATGGACTTACCAGCCTTGA-3'  5'-GAGGCGGACCTTGGAAAGTTTTA-3'  5'-GGGTTTCACGCAGTTTCAGCAGT-3'  5'-AAGGAGGGGAGGTCAATTTTCTT-3'  5'-TGGCATCACACTTTCTACAA -3'  5'-CAACGGAATCTCTCAGCTCC-3' | 210  215  220  240  242  250  238  151  176  194  203  198  217  176  218  184  248  246  205 | 55  56  56  55  54  58  58  58  59  54  58  55  54  56  56  54  54  56  55 |
